# Intrachromosomal karyotype asymmetry in Orchidaceae

**DOI:** 10.1590/1678-4685-GMB-2016-0264

**Published:** 2017-06-22

**Authors:** Enoque Medeiros-Neto, Felipe Nollet, Ana Paula Moraes, Leonardo P. Felix

**Affiliations:** 1 Departamento de Ciências Biológicas, Laboratório de Citogenética Vegetal, Centro de Ciências Agrárias, Universidade Federal da Paraíba, Campus II, Areia, PB, Brazil; 2 Departamento de Genética, Instituto de Biociências, Universidade Estadual Paulista “Júlio de Mesquita Filho”, Botucatu, SP, Brazil; 3 Instituto de Ciências e Tecnologia, Universidade Federal de São Paulo, São José dos Campos, SP, Brazil

**Keywords:** Asymmetry index, karyotype symmetry, chromosome, cytogenetic, evolution

## Abstract

The asymmetry indexes have helped cytotaxonomists to interpret and classify plant karyotypes for species delimitation efforts. However, there is no consensus about the best method to calculate the intrachromosomal asymmetry. The present study aimed to compare different intrachromosomal asymmetry indexes in order to indicate which are more efficient for the estimation of asymmetry in different groups of orchids. Besides, we aimed to compare our results with the Orchidaceae phylogenetic proposal to test the hypothesis of Stebbins (1971). Through a literature review, karyotypes were selected and analyzed comparatively with ideal karyotypes in a cluster analysis. All karyotypes showed some level of interchromosomal asymmetry, ranging from slightly asymmetric to moderately asymmetric. The five tested intrachromosomal asymmetry indexes indicated *Sarcoglottis grandiflora* as the species with the most symmetrical karyotype and *Christensonella pachyphylla* with the most asymmetrical karyotype. In the cluster analysis, the largest number of species were grouped with the intermediary ideal karyotypes B or C. Considering our results, we recommend the combined use of at least two indexes, especially Ask% or A1 with Syi, for cytotaxonomic analysis in groups of orchids. In an evolutionary perspective, our results support Stebbins’ hypothesis that asymmetric karyotypes derive from a symmetric karyotypes.

## Introduction

The karyotype is the first phenotypic expression of the genotype and provides an overview of the organization of the genetic material in the chromosome (Guerra, 2008). Among the information that can be extracted from karyotypes, *i.e.* number and morphology of the chromosomes, diversity of heterochromatic bands, gene location, etc., a very peculiar characteristic stands out: the karyotype asymmetry, which is the subject of long debates. Changes in karyotype symmetry often involve modifications in chromosome size and morphology usually caused by DNA sequence expansions or deletions or by centric fusion/fissions (accompanied by disploidy) (Weiss-Schneeweiss and Schneeweiss, 2013).

The search for an index that reflected the karyotype asymmetry started with Lewitsky (1931) and was followed by Huziwara (1962), Arano (1963), Stebbins (1971), and many other authors (for a detailed discussion see Peruzzi and Eroğlu, 2013). For a long time, these indexes were employed by many cytogeneticists and cytotaxonomists to discuss the taxonomic relationships among related species (Dematteis, 1998; D’Emerico *et al.*, 1999; Selvi *et al.*, 2006; Felix *et al.*, 2007; Peruzzi *et al.*, 2009; Souza *et al.*, 2010). Stebbins (1971) suggested that asymmetric karyotypes were originated from symmetrical ones, which has not been properly tested until now.

The existing indexes are separated into two groups: interchromosomal asymmetry indexes, which quantify the heterogeneity in chromosome size, and intrachromosomal asymmetry indexes, which quantify the relative differences in the centromere position among chromosomes of a complement (Stebbins, 1971; Peruzzi and Eroğlu, 2013). Among the interchromosomal asymmetry indexes, A_2_ (Romero-Zarco, 1986) and CV_CL_ (Paszko, 2006) are the most used due to their accuracy in the evaluation of chromosome dissimilarities (Chiarini and Barboza, 2008; Souza *et al.*, 2010; Pierozzi, 2011; Alves *et al.*, 2011; Assis *et al.*, 2013). However, there is no consensus about the best method for calculating the intrachromosomal asymmetry (Romero-Zarco, 1986; Peruzzi and Eroğlu, 2013).

Among the proposed intrachromosomal asymmetry indexes, the following stand out:

*The four categories of* *Stebbins* (1971): from A to D according to the proportion of acrocentric and/or telocentric chromosomes in a karyotype, *i.e.* proportion of chromosomes with a ratio between chromosome arms < 2:1. The four categories have subtypes 1 to 3 according to the ratio between the large/small chromosome arms, giving a total of 12 categories (Table 1).

**Table 1:** Intrachromosomal asymmetry indexes. The total of 12 indexes is composted of the four categories of Stebbin*s* (1971) - A to D according to the proportion of acrocentric and/or telocentric chromosomes in a karyotype - and subtypes 1 to 3 according to the ratio between the large/small chromosome arms in each of these.

Ratio: largest/smallest chromosomes	Proportion of chromosomes with arm ratio < 2:1			
	0.0	0.01 – 0.5	0.51 – 0.99	1.0
< 2 : 1	1 A	1 B	1 C	1 D
2:1 – 4:1	2 A	2 B	2 C	2 D
> 4:1	3 A	3 B	3 C	3 D

*The total form percentage* (TF%; Huziwara, 1962): ratio between the sum of the short arms (p) length and the sum of the total chromosomes length:

TF%=∑p/∑total karyotype length

*The karyotype asymmetry index percentage* (Ask%; Arano, 1963): ratio between the length of the long arms (q) of the chromosome set and the total length of the chromosome set:

Ask%=∑q/∑total karyotype length

*The symmetric index* (Syi; Greilhuber and Speta, 1976): ratio between the average length of the short arms (p) and the average length of the long arms, multiplied by 100:

Syi=(∑mean of p length/∑mean of q length)×100

*The intrachromosomal asymmetry index A*_*1*_ (Romero-Zarco 1986): sum of the ratio between the average length of the short arms in each homologous pairs (*b*_*i*_) and the average length of the long arms in each homologous pair (*B*_*i*_) divided by the number of homologous chromosome pairs (*n*):

$$A_{1}=1-\frac{{\text{∑}}_{i=1}^{n}\left(b_{i}/B_{i}\right)}{n}$$

This proposal was later modified by Watanabe *et al.* (1999), who created the *asymmetry index A*, and followed by Peruzzi and Eroğlu (2013), who called the same index M_CA._ This index results from the sum of the ratio between the differences in the long arm length (*B*_*i*_) and the short arm length (*b*_*i*_) of each chromosome and the sum of the lengths of the long and short arms of each chromosome (*B*_*i*_ *+ b*_*i*_). The sum is divided by the haploid chromosome number (*n*):

$$A=\frac{{\text{∑}}_{i=1}^{n}\left(B_{i}-b_{i}/B_{i}+b_{i}\right)}{n}$$

*The coefficient of variation of the centromeric index* CV_CI_ (Paszko, 2006): based on the index of interchromosomal asymmetry A2 (standard deviation/total average of the chromosome length):

$$CV_{\text{CI}}=A_{2}\times 100$$

With so many ways to calculate the intrachromosomal asymmetry, Zuo and Yuan (2011) developed six models of ideal karyotypes to test the accuracy of these methods. These authors observed that the CV_CI_ does not reflect the intrachromosomal asymmetry in karyotypes when it is composed by telocentric and/or acrocentric chromosomes. Since the standard deviation decreases in acrocentric or telocentric chromosomes, the CV_CI_ is more suitable to indicate the heterogeneity of the centromeric index, *i.e.* how different is the position of the centromeres among the chromosomes of the complement (see Zuo and Yuan, 2011).

Peruzzi and Eroğlu (2013) discouraged the use of any intrachromosomal asymmetry index for karyotypes with small chromosomes (≤ 1 μm), due to the inaccuracy in the arms’ measurement. However, the mean chromosome size in plants is 1.5–2.0 μm. Moreover, if the suggestion of Peruzzi and Eroğlu (2013) is followed, the chromosome symmetry analysis will be prohibitive in a large number of plant species, including a great part of Bromeliaceae, Fabaceae and Orchidaceae species.

Considered one of the most diverse and taxonomically complex plant families among the angiosperms, Orchidaceae comprises 25,971 species with global distribution (Pridgeon *et al.*, 1999; Joppa *et al.*, 2011). The Orchidaceae present a large karyotype variation, with all types of chromosome morphology distributed in species with chromosome numbers varying from 2*n* = 12 in *Erycina pusilla* (L.) N.H.Williams & M.W.Chase (Felix and Guerra, 1999) to 2*n* = 240 in *Epidendrum cinnabarinum* Salzm. ex Lindl. (Guerra, 2000; Felix and Guerra, 2010; Assis *et al.*, 2013). Except for the subfamily Cypripedioideae, Orchidaceae species are characterized by small chromosomes (Larsen, 1968; Okada, 1988).

The wide karyotype diversity observed in Orchidaceae makes this plant family an excellent group for evaluating the applicability of karyotype asymmetry indexes, especially the intrachromosomal index. Thus, the present study aimed to compare different intrachromosomal asymmetry indexes, in order to indicate the most efficient for the estimation of karyotype asymmetry in orchids, including species with small chromosomes. Besides, we aimed to compare our results with the Orchidaceae phylogenetic proposal to test the hypothesis of Stebbins (1971) that asymmetric karyotypes derived from symmetric ones.

## Material and Methods

### Chromosome measurements

A literature search was performed to select informative photographic records of metaphases quality – clear identification of centromere and secondary constrictions – and the available voucher (Table 2).

**Table 2:** Karyomorphometric data for the representatives of the family Orchidaceae and respective references, with diploid chromosome number (2*n*), karyotype formula, chromosome size variation (μm), total sum of the haploid chromosome length (hcl), the interchromosomal index (Inter Index) by Romero-Zarco (A2), and the intrachromosomal indexes: Huziwara index (TF%), Arano index (Ask%), Greilhuber and Speta index (Syi), Romero-Zarco (A_1_) and Watanabe index (A). The bold and underlined numbers indicate the most symmetrical and asymmetric karyotypes for each index, respectively.

Taxa	References*	2*n*	Karyotype formula	Chromosome size variation (μm)	hcl	Inter Index	Intrachromosomal Index				
						A_2_	TF%	Ask%	Syi	A1	A
Cypripedioideae											
*Paphiopedilum dianthum* Tang & F.T.Wang	LA11	26	18M+8S	9.34 – 22.22	395.88	0.23	0.41	0.59	69.06	0.30	0.18
*P. emersonii* Koop. & P.J.Cribb	LA11	26	20M+4S+2A	5.06 – 12.56	204.98	0.24	0.40	0.60	66.36	0.31	0.20
*P. hangianum* Perner & O.Gruss	LA11	26	22M+4S	9.66 – 18.11	325.92	0.20	0.44	0.56	80.04	0.18	0.11
*P. micranthum* Tang & F.T.Wang	LA11	26	16M+8S+2A	7.27 – 22.47	296.44	0.35	0.40	0.60	66.19	0.28	0.20
*P. niveum* (Rchb.f.) Stein	LA11	26	20M+6S	6.46 – 18.35	255.90	0.27	0.45	0.55	80.99	0.19	0.11
*P. randsii* Fowlie	LA11	26	18M+8S	10.74 – 22.48	377.84	0.24	0.44	0.56	78.63	0.21	0.12
*P. sukhakulii* Schoser & Senghas	LA11	40	24M+16S	6.42 – 18.84	391.64	0.31	0.41	0.59	69.99	0.30	0.18
*P. superbiens* (Rchb.f.) Stein [as *P. curtisii* (Rchb.f.) Stein]	LA11	36	22M+14S	7.33 – 20.78	385.86	0.30	0.40	0.60	68.06	0.30	0.19
*Phragmipedium sargentianum* (Rolfe) Rolfe	FG05	22	14M+8S	2.94 – 6.49	100.24	0.28	0.42	0.58	73.07	0.27	0.16
Epidendroideae											
*Acianthera recurva* (Lindl.) Pridgeon & M.W.Chase	OL15	52	38M+14S	1.08 – 2.05	76.78	0.15	0.44	0.56	77.20	0.22	0.13
*Brasiliorchis gracilis* (Lodd., G.Lodd. & W.Lodd.) R.B.Singer, S.Koehler & Carnevali	MO12	40	19M+21S	2.39 – 4.87	148.92	0.18	0.40	0.60	66.81	0.33	0.20
*Brassavola nodosa* (L.) Lindl.	FG10	40	16M+24S	1.06 – 2.11	63.52	0.15	0.39	0.61	64.24	0.35	0.22
*Campylocentrum crassirhizum* Hoehne	FG10	38	12M+24S+2A	0.87 – 1.56	48.56	0.13	0.37	0.63	57.53	0.41	0.27
*C. neglectum* (Rchb.f. & Warm.) Cogn.	DA09	38	20M+18S	0.62 – 1.20	33.64	0.19	0.40	0.60	65.91	0.33	0.21
*Catasetum purum* Nees & Sinning	FG00	54	32M+22S	0.93 – 3.09	93.10	0.25	0.41	0.59	69.53	0.29	0.18
*Cattleya bradei* (Pabst) Van den Berg [as *Hoffmannseggella bradei* (Pabst) V.P.Castro & Chiron]	Y06	40	24M+16S	1.09 – 2.53	75.70	0.16	0.40	0.60	67.40	0.32	0.19
*C. cernua* (Lindl.) Van den Berg [as *Sophronitis cernua* (Lindl.) Lindl.]	DA09	40	18M+16S+6A	0.92 – 2.41	63.14	0.25	0.36	0.64	56.25	0.39	0.28
*Cephalanthera damasonium* (Mill.) Druce	MS07	36	14M+14S+8A	2.46 – 16.0	219.36	0.60	0.29	0.71	40.32	0.44	0.43
*C. longifolia* (L.) Fritsch	MS07	32	12M+16S+4A	2.68 – 9.82	146.90	0.47	0.33	0.67	50.13	0.40	0.33
*C. rubra* (L.) Rich.	MS07	44	14M+30S	2.43 – 12.52	230.94	0.49	0.36	0.64	57.07	0.38	0.27
*Christensonella pachyphylla* (Schltr. ex Hoehne) Szlach. (as *Maxillaria madida var. Monophylla* Cogn.)	K08	38	20S+18A	2.17 – 4.35	119.86	0.17	0.26	0.74	34.36	0.64	0.49
*C. paranaensis* (Barb.Rodr.) S.Koehler (as *Maxillaria heterophylla var. Pygmaea* Hoehne)	K08	36	20M+16S	1.92 – 3.31	95.28	0.13	0.40	0.60	68.02	0.30	0.19
*C. pumila* (Hook.) Szlach.	K08	36	16M+18S+2A	2.02 – 3.38	97.78	**0.12**	0.37	0.63	59.49	0.37	0.25
*C. subulata* (Lindl.) Szlach. [as *C. acicularis* (Herb. ex Lindl.) Szlach.]	K08	38	4M+20S+14A	1.90 – 3.34	92.80	0.14	0.28	0.72	39.23	0.59	0.44
*Dimerandra emarginata* (G.Mey.) Hoehne	FG10	40	28M+12S	0.86 – 2.14	60.60	0.20	0.43	0.57	75.47	0.24	0.14
*Encyclia flava* (Lindl.) Porto & Brade	FG10	40	10M+28S+2A	1.31 – 3.59	90.54	0.22	0.37	0.63	58.12	0.42	0.26
*E. oncidioides* (Lindl.) Schltr.	FG10	40	16M+22S+2A	1.28 – 2.71	75.38	0.16	0.37	0.63	59.87	0.39	0.25
*Epidendrum denticulatum* Barb.Rodr.	AS13	38	28M+6S+4A	1.0 – 3.05	65.46	0.26	0.43	0.57	75.47	0.24	0.14
*E. fulgens* Brongn.	AS13	24	14M+8S+2A	1.10 – 3.01	36.26	0.29	0.40	0.60	68.03	0.32	0.18
*E. latilabre* Lindl.	FG10	40	6M+34S	1.10 – 2.10	62.62	0.13	0.36	0.64	55.69	0.44	0.28
*E. paniculatum* Ruiz & Pav.	AS13	40	20M+16S+4A	1.08 – 2.26	66.48	0.17	0.39	0.61	64.29	0.34	0.22
*Erycina pusilla* (L.) N.H.Williams & M.W.Chase [as *Psygmorchis pusilla* (L.) Dodson & Dressler]	FG99	12	8M+4A	2.85 – 5.46	45.90	0.21	0.34	0.66	52.21	0.39	0.31
*Mormolyca rufescens* (Lindl.) M.A.Blanco (as *Maxillaria rufescens* Lindl.)	FG00	40	30M+10S	1.04 – 2.02	62.98	0.18	0.42	0.58	73.55	0.25	0.15
*Pabstiella fusca* (Lindl.) Chiron & Xim.Bols.	OL15	28	14M+14S	0.62 – 1.32	27.78	0.17	0.39	0.61	65.16	0.32	0.21
*Prosthechea fragrans* (Sw.) W.E.Higgins	FG10	40	18M+22S	1.04 – 2.06	55.82	0.15	0.39	0.61	64.32	0.35	0.22
*Sobralia liliastrum* Lindl.	FG10	48	34M+14S	1.51 – 4.15	109.52	0.27	0.43	0.57	74.20	0.25	0.15
*Specklinia grobyi* (Bateman ex Lindl.) F.Barros	OL15	20	18M+2A	0.88 – 2.07	30.66	0.20	0.45	0.55	80.57	0.20	0.11
*Stelis sp.*	FG10	32	12M+20S	0.73 – 1.35	29.38	0.18	0.38	0.62	60.40	0.38	0.25
*Trichocentrum cebolleta* (Jacq.) M.W.Chase & N.H.Williams [as *Oncidium cebolleta* (Jacq.) Sw.]	FG00	36	20M+14S+2A	1.79 – 2.91	79.38	0.14	0.40	0.60	66.31	0.31	0.20
*T. fuscum* Lindl. (as *T. cornucopiae* Linden & Rchb.f.)	FG00	20	8M+12S	2.38 – 4.87	73.46	0.21	0.39	0.61	64.80	0.33	0.21
*T. pumilum* (Lindl.) M.W.Chase & N.H.Williams (as *Oncidium pumilum* Lindl.)	FG00	30	19M+11S	2.47 – 6.75	128.94	0.25	0.41	0.59	70.45	0.28	0.17
*Xylobium foveatum* (Lindl.) G.Nicholson	FG00	40	26M+14S	1.84 – 3.88	114.84	0.20	0.41	0.59	70.64	0.28	0.17
*Zygostates alleniana* Kraenzl.	DA09	54	36M+18S	1.0 – 2.72	80.92	0.27	0.41	0.59	70.93	0.26	0.17
Orchidoideae											
*Aspidogyne kuczynskii* (Porsch) Garay	DA09	42	24M+18S	0.64 – 1.46	45.24	0.15	0.41	0.59	69.69	0.29	0.18
*Cyclopogon calophyllus* (Barb.Rodr.) Barb.Rodr.	GR13	28	22M+6S	1.87 – 4.01	70.50	0.20	0.43	0.57	76.09	0.23	0.14
*C. chloroleucus* Barb.Rodr.	FG05	36	32M+4S	1.50 – 4.80	91.30	0.30	0.44	0.56	79.12	0.19	0.12
*C. congestus* (Vell.) Hoehne [as *Beadlea congesta* (Vell.) Garay]	MA84	32	8M+24S	0.86 – 2.77	56.56	0.28	0.37	0.63	58.05	0.42	0.27
*C. elatus* (Sw.) Schltr.	FG05	28	24M+4S	1.69 – 3.39	65.32	0.18	0.44	0.56	78.28	0.21	0.12
*C. elatus* (Sw.) Schltr.	GR13	28	18M+10S	1.87 – 4.26	75.34	0.19	0.43	0.57	75.49	0.23	0.14
*Eltroplectris calcarata* (Sw.) Garay & H.R.Sweet	MN16	42	22M+18S+2A	1.93 – 9.79	122.26	0.53	0.35	0.65	54.96	0.36	0.29
*E. actinosophila* (Barb.Rodr.) Schltr.	DA09	56	32M+20S+4A	1.04 – 2.02	77.32	0.17	0.40	0.60	66.32	0.32	0.20
*Eurystyles sp.*	MN16	14	12M+2S	1.63 – 3.17	31.40	0.23	0.45	0.55	80.98	0.19	0.11
*Habenaria bicornis* Lindl.	BA14	42	22M+20S	3.63 – 6.83	214.24	0.13	0.41	0.59	68.18	0.31	0.19
*H. josephensis* Barb.Rodr.	MN16	50	34M+2S+14A	1.67 – 4.97	161.44	0.29	0.41	0.59	68.68	0.33	0.19
*Mesadenella cuspidata* (Lindl.) Garay	MN16	46	32M+12S+2A	1.86 – 4.38	106.46	0.21	0.40	0.60	68.05	0.28	0.19
*Pelexia laxa* (Poepp. & Endl.) Lindl.	MA84	46	18M+28S+2A	0.88 – 2.63	56.98	0.27	0.36	0.64	56.20	0.41	0.28
*P. viridis* (Cogn.) Schltr.	FG05	46	36M+8S+2A	1.30 – 4.72	92.52	0.30	0.41	0.59	70.62	0.25	0.17
*Prescottia plantaginea* Lindl.	MN16	46	42M+4S	1.20 – 2.66	82.38	0.17	0.45	0.55	82.31	0.17	0.10
*Pteroglossa lurida* (M.N.Correa) Garay [as *Eltroplectris lurida* (M.N.Correa) Pabst]	MA84	46	12M+32S+2A	0.75 – 4.32	52.76	0.60	0.33	0.67	49.59	0.43	0.34
*Sacoila lanceolata* (Aubl.) Garay	MN16	46	40M+4S+2A	1.65 – 8.34	109.02	0.46	0.41	0.59	70.73	0.21	0.17
*Sarcoglottis grandiflora* (Hook.) Klotzsch	MN16	46	44M+2S	1.42 – 3.49	85.12	0.21	0.47	0.53	87.37	0.11	0.07
Vanillioideae											
*Vanilla pompona* Schiede	FG05	32	10M+20S+2A	1.04 – 3.36	63.26	0.26	0.37	0.63	57.88	0.39	0.27

* AS13 = Assis *et al.* (2013); BA14 = Batista *et al.* (2014); DA09 = Daviña *et al.* (2009); FG99 = Felix and Guerra (1999); FG00 = Felix and Guerra (2000); FG05 = Felix and Guerra (2005); FG10 = Felix and Guerra (2010); GR13 = Grabiele *et al.* (2013); K08 = Koehler *et al.* (2008); LA11 = Lan and Albert (2011); MA84 = Martinez (1984); MN16 = Medeiros-Neto (unpublished data); MO12 = Moraes *et al.* (2012); MS07 = Moscone *et al.* (2007); OL15 = Oliveira *et al.* (2015); Y06 = Yamagishi (2006, Doctoral Thesis, Universidade Estadual de Campinas, Campinas, SP, Brazil).

The arm ratio (r = length of the long arm/length of the short arm) was used to classify the chromosomes as metacentric (M: r = 1.00 to 1.49), submetacentric (S: r = 1.50 to 2.99), acrocentric (A: r ≥ 3.00) and telocentric (T: r = ∞), according to Guerra (1986). We did not consider differences between acrocentric and telocentric chromosomes. For chromosome measurements we used Imagetool^®^ software version 3.0 (available at http://compdent.uthscsa.edu/dig/itdesc.html) calibrated with scales available on the selected images.

The interchromosomal asymmetry was calculated using A_2_ by Romero-Zarco (1986). Five different indexes were estimated for the intrachromosomal asymmetry: TF% (Huziwara, 1962); Ask% (Arano, 1963); Syi (Greihuber and Speta, 1976); A_1_ (Romero-Zarco, 1986); and A (Watanabe *et al.*, 1999). In addition, the ideal karyotypes (Zuo and Yuan, 2011) were used to establish a comparative standard for the analyzed karyotypes. The ideal karyotypes, ranked from A to F, present chromosome morphology based on Levan *et al.* (1964). However, here we followed the nomenclature proposed by Guerra (1986) based on the percentage of acrocentric/telocentric chromosomes: (1) up to 30% (slightly asymmetric - SA); (2) from 31 to 50% (moderately asymmetric - MA) and (3) more than 50% (strongly asymmetric - StA).

### Cluster analysis

The intrachromosomal index values were separately used for cluster analysis. The obtained values were categorized and used to define the limits of each category (Table S1). For the cluster analysis we used the UPGMA algorithm (unweighted pair-group method with arithmetic means) implemented in the software Mesquite^®^ (Maddison and Maddison, 2015). Ten trees were generated for each index using the distances from the data matrix by majority consensus. Subsequently, only one consensus tree was stored. The software Dendroscope^®^ (Huson and Scornavacca, 2012) was used to root the tree with the most ideal symmetrical karyotype (karyotype A) as an outgroup, following the hypothesis of Stebbins (1971).

### Statistical analysis

To test the hypothesis of Stebbins (1971), the mean values of the interchromosomal index A2 and the intrachromosomal asymmetry indexes were separately used, in order to compare the karyotype asymmetry levels for each subfamily. The variation between the mean values of asymmetry indexes for the subfamilies was compared statistically by ANOVA followed by Tukey's test using BioEstat v.5.3 (Ayres *et al.*, 2007).

## Results

### Karyomorphometric analysis

The metaphases of 64 species, distributed throughout four subfamilies (Cypripedoideae, Epidendroideae, Orchidoideae and Vanilloideae) were selected across 16 references (Table 2). No karyotype analysis was found for Apostasioideae. Chromosome numbers ranged from 2*n* = 12 in *Erycina pusilla* (Epidendroideae) to 2*n* = 56 in *Eurystyles actinosophila* (Barb. Rodr.) Schltr (Orchidoideae). The *Campylocentrum neglectum* (Rchb.f. & Warm.) Cogn. (Epidendroideae) presented the smallest chromosomes, ranging from 0.62 μm to 1.20 μm, while *Paphiopedilum randsii* Fowlie (Cypripedioideae) presented the largest chromosomes, ranging from 10.74 μm to 22.48 μm (Table 2). The total sum of haploid chromosome length (hcl) ranged from 27.78 μm in *Pabstiella fusca* (Lindl.) Chiron & Xim.Bols. (Epidendroideae) to 395.88 μm in *Paphiopedilum dianthum* Tang & F.T.Wang (Cypripedioideae) (Table 2).

**Figure 1:**
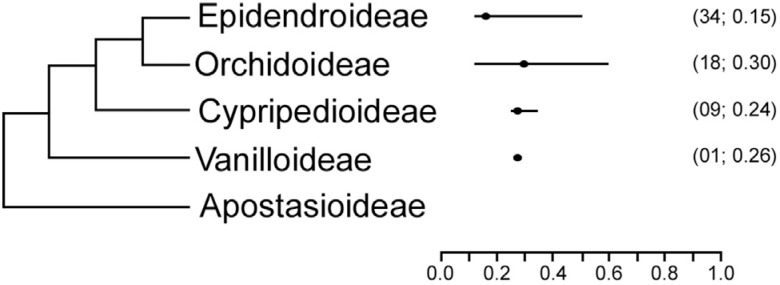
Interchromosomal index A2 values for Orchidaceae subfamily. For each subfamily the mean value (dot), the amplitude of variation (bar), the number of species analyzed and mode value (last two data in the parenthesis, respectively) are presented.

Regarding interchromosomal asymmetry (A_2_), the most symmetric karyotype was found in *Christensonella pumila* (Hook.) Szlach. (A_2_ = 0.12), while the most asymmetrical were found in *Cephalanthera damasonium* (Mill.) Druce and *Pteroglossa lurida* (M.N.Correa) Garay (A_2_ = 0.60; see Table 2). However, when comparing the subfamilies, the statistical test failed to show any difference (F = 1.6526, p = 0.1988; Figure 1).

The five tested intrachromosomal asymmetry indexes provided the same result: the most symmetric karyotype was found in *Sarcoglottis grandiflora* (Hook.) Klotzsch (44M + 2S; see bold numbers in Table 2) and the most asymmetric karyotype was found in *Christensonella pachyphylla* (Schltr. ex Hoehne) Szlach., (20S + 18A; see underlined numbers in Table 2).

### Cluster analysis for intrachromosomal indexes

**Figure 2:**
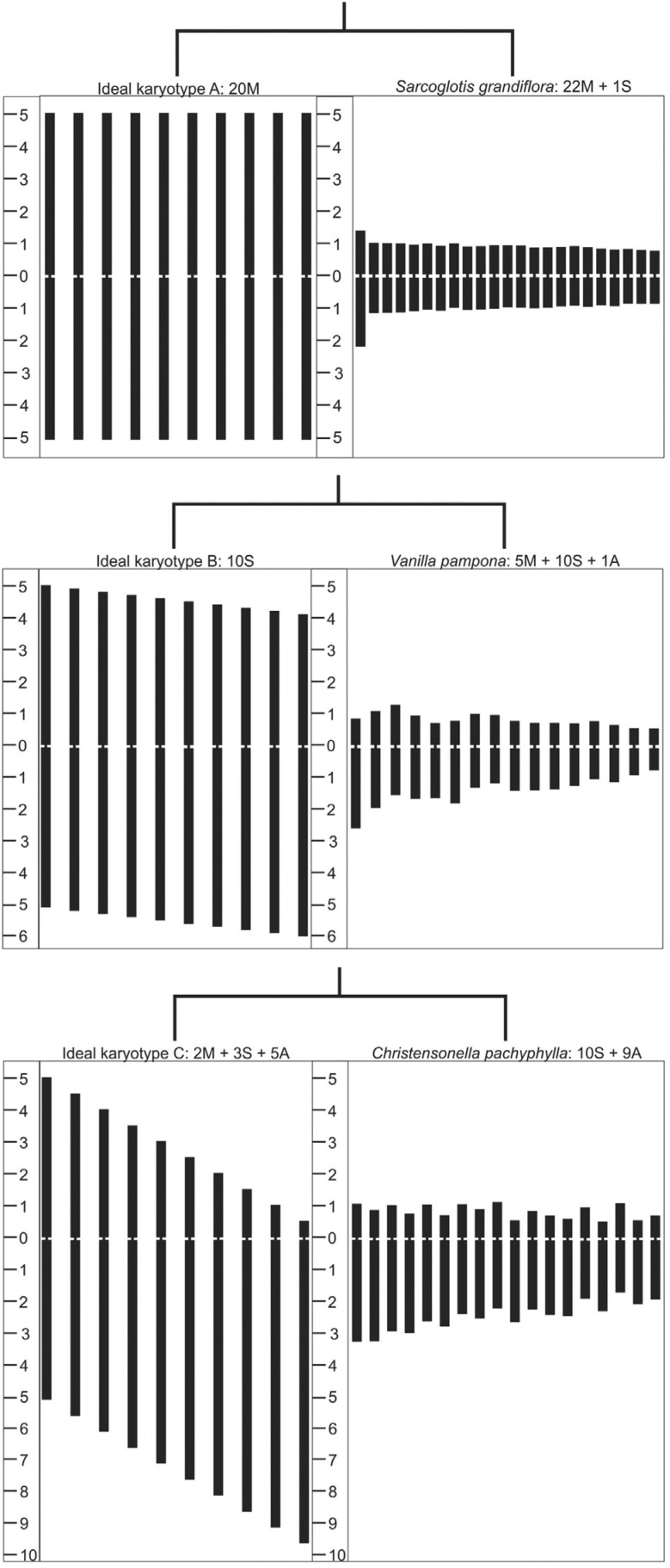
Ideograms of the ideal karyotypes A, B and C, as well as the most similar species, grouped by UPGMA, equally obtained by the indexes Ask%, A1, Syi and A. The numeric scale at the right side of the ideogram is given in micrometers (μm).

Cluster analysis using the values found for TF% grouped most species with the ideal karyotype C, the *Sarcoglottis grandiflora* with the ideal karyotype B and *Christensonella pachyphylla* with the ideal karyotype D (Figure S1). Ask% and A_1_ indexes presented identical trees (Figure S2) in the cluster analysis, with most species grouped with ideal karyotype B, plus a clade, separated into two groups: (1) a polytomy with the ideal karyotypes D, E and F and (2) a group with *Christensonella pachyphylla, C. subulata* (Lindl.) Szlach. and the ideal karyotype C. The cluster analysis for A and Syi also formed identical trees (Figure S3), similar to trees obtained with Ask% and A1 (Figure S2). A difference was found with *Christensonella subulata,* which was grouped with most species and the ideal karyotype B (instead of C). The indexes Ask% and A1 (Figure S2), and Syi and A (Figure S3) grouped *Sarcoglottis grandiflora* as a sister group of karyotype A. The indexes Ask%, A1, Syi and A provided more consistent groups (Figure 2), reflecting the species karyotype composition in the ideal karyotypes, as proposed by Zuo and Yuan (2011).

**Figure 3:**
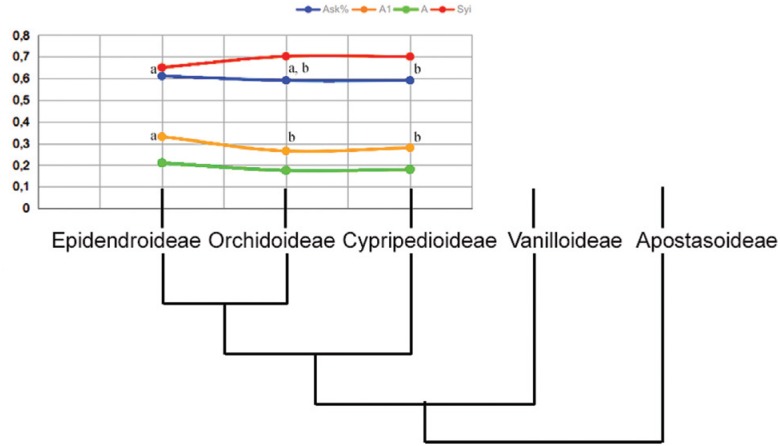
Intrachromosomal asymmetry values obtained by Ask% (blue), A1 (red), A (green) and Syi (orange) indexes for the Orchidaceae subfamily. The numeric scale at the right side indicates the mean value for the four intrachromosomal indexes. The Syi value was divided by 100. Subfamilies indicated by the same letters are not significantly different (Tukey test, p < 0.05).

Comparing the four most congruent intrachromosomal indexes, Ask%, A1, Syi and A, with the current proposed Orchidaceae phylogeny (Chase *et al.*, 2015), all indexes presented similar mean and mode values for Orchidoideae and Cypripedioideae (Figure 3). Index A did not detected a difference among subfamilies, after Tuckey's test (despite the F = 4.1420, p = 0.0203). The indexes Ask% and Syi indicated that karyotypes from Epidendroideae and Orchidoideae are more asymmetrical than Cypripedioideae - the most basal subfamily among the three (F = 4.4915 and 4.7008, respectively; p = 0.01 for both indexes). The A1 index suggested Epidendroideae as the most asymmetric karyotype among subfamilies (F = 5.77, p = 0.0054).

## Discussion

The inter and intrachromosomal asymmetry values observed here corroborate previous studies, with a slight variation for some species, such as *Epidendrum paniculatum* Ruiz & Pav., *E. fulgens* Brongn. (Assis *et al.*, 2013), *Cyclopogon calophyllus* (Barb.Rodr.) Barb.Rodr. and *C. elatus* (Sw.) Schltr. (Grabiele *et al.*, 2013). Therefore, we can observe that the relationship between the two kinds of asymmetry (intra and interchromosomal) is not always unidirectional, but it is a result of complex rearrangements that modify both the centromere position and the chromosome size in a karyotype.

### The interchromosomal index

The A_2_ index employed here yielded values close to zero for some species, mainly in the subfamilies Epidendroideae and Orchidoideae. In such cases, the index reflects a conservation among chromosome size in the karyotype; other species, however, presented high A_2_ values.

The highly asymmetric karyotypes could be the result of chromosome rearrangements, what could also cause bimodality, as observed in *Cephalanthera damasonium* (Epidendroideae; Moscone *et al.*, 2007) and *Pteroglossa lurida* (Orchidoideae; Martinez 1984), both with A_2_ = 0.60. The origin of bimodal karyotypes could be due to the loss of chromosome segments after polyploidy, resulting in the formation of smaller chromosomes (Weiss-Schneeweiss and Schneeweiss, 2013), or due to unequal translocations (Stebbins, 1971), differential amplification of heterochromatic regions (de la Herrán *et al.*, 2001), or even in the hybridization between species with different chromosome sizes. All these events increase the interchromosomal asymmetry by increasing the morphological discontinuities between chromosomes in a karyotype.

### The intrachromosomal indexes

Regarding the intrachromosomal asymmetry, we showed that Orchidaceae karyotypes ranged from slightly asymmetric to moderately asymmetric. The intrachromosomal asymmetry is defined by the presence of a greater number of acrocentric/telocentric chromosomes in relation to the metacentric and submetacentric ones, a consequence of changes in centromere position (Stebbins, 1971) – in which case the chromosome rearrangement could affect all chromosomes in the same way and even increase the karyotype asymmetry. Therefore, the efficacy of the intrachromosomal asymmetry indexes is dependent on the precise identification of the centromere and a well-defined chromosome morphology, and not on chromosome size. The indexes Ask% and A_1_ proved to be more useful in determining the intrachromosomal asymmetry, even in species with small chromosomes, like *Campylocentrum neglectum*.

The extreme symmetry (ideal karyotype A) or the extreme asymmetry (ideal karyotype F) karyotypes are hardly found in nature. However, in the present analysis, an extreme of symmetric karyotype was found in *Sarcoglottis grandiflora*, grouped with the ideal karyotype A. *Christensonella pachyphylla* showed the most asymmetric karyotype, but this species was grouped with ideal karyotypes C and D and not with the extreme ideal karyotype F.

The occurrence of asymmetric karyotypes is probably a consequence of chromosomal structural changes, especially centric fusion/fission, a very common rearrangement in Orchidaceae (Pinheiro *et al.*, 2009; Yamagishi-Costa and Forni-Martins, 2009; Assis *et al.*, 2013; Moraes *et al.*, 2012, 2013; 2016). *Christensonella* Szlach. presents a dysploidy variation (2*n* = 36 and 38) and the occurrence of centric fusion/fission is suggested as the main cause. This is illustrated by the double DAPI^+^ band holding the centromere in *C. fernandiana*, as the remainder species present just one DAPI^+^ band (Koehler *et al.*, 2008; Moraes *et al.*, 2012). The centric fusion/fission is suggested to be the cause of the frequent dysploidy in other genera in subtribe Maxillariinae (Moraes *et al.*, 2012; 2016) and Orchidaceae (Pinheiro *et al.*, 2009; Felix and Guerra, 2010; Assis *et al.*, 2013). The same can be detected in other plant groups as the genus *Tristagma* Poepp. (as *Ipheion* Raf., Souza *et al.*, 2010): *Tristagma tweedieanum* (Baker) Traub (2*n* = 14, karyotypic formula 14A, A_1_ = 0.86) and *T. uniflorum* (Lindl.) Traub (2*n* = 12, karyotypic formula = 2SM + 10A, A_1_ = 0.78), family Iridaceae (Goldblatt, 1990; Alves *et al.*, 2011; Moraes *et al.*, 2015), Asteraceae (*Brachyscome* Cass.; Watanabe *et al.*, 1999) and Sapindaceae (*Serjania* Mill.; Coulleri *et al.*, 2012).

### The Stebbins’ hypothesis

The relationship between karyotype asymmetry and species evolution could be discussed based on intrachromosomal indexes, since the interchromosomal index does not differ among subfamilies. The intrachromosomal asymmetry indexes indicated the karyotypes of some representatives of the subfamily Epidendroideae as the most asymmetric – in agreement with the hypothesis of Stebbins (1971) that asymmetric karyotypes had been originated from symmetrical ones. Based on the statistical results and cluster analysis the congruent indexes Ask%, A1 and Syi indicated Epidendroideae as the most derivate subfamily, presenting the most asymmetrical karyotype, while the representatives of the subfamily Cypripedioideae have more symmetrical karyotypes.

## Conclusions

Considering our results, the indexes Ask% (Arano, 1963), A_1_ (Romero-Zarco, 1986) and Syi are recommended for the estimation of intrachromosomal asymmetry in cytotaxonomic studies, especially in a combined fashion. We showed that the critical point for the efficacy of an asymmetric index is the well-preserved chromosome morphology and precise definition of the centromere position – and not the size of chromosomes. Moreover, the higher karyotype asymmetry associated with the derivative subfamily Epidendroideae supports Stebbins’ hypothesis that asymmetric karyotypes tend to derive from symmetric karyotypes.

## Supplementary Material

The following online material is available for this article:

Table S1 - Asymmetry values for ideal karyotypes.

Figure S1 - UPGMA analysis using the intrachromosomal asymmetry values from TF% index.

Figure S2 - UPGMA analysis using the intrachromosomal asymmetry values from Ask% and A_1_ indexes.

Figure S3 - UPGMA analysis using the intrachromosomal asymmetry values from Syi and A indexes.
