# Supplementary material for: Intrachromosomal karyotype asymmetry in Orchidaceae
Source: Genet Mol Biol. 2017 Jun 22;40(3):610–9. doi: 10.1590/1678-4685-GMB-2016-0264 (PMC5596371; doi:10.1590/1678-4685-GMB-2016-0264)
Supplement: Supplementary file 1 [file 1415-4757-gmb-1678-4685-GMB-2016-0264-Suppl01.pdf]

Table S1 - Value of chromosome asymmetrical indexes adapted for ideal karyotypes following Zuo and Yuan (2011).

Ideal karyotype	Karyotypic Formula*	Huziwara (1962) TF%	Arano (1963) Ask%	Greilhuber and Speta (1976) Syi	Romero-Zarco (1986) A_1	Watanabe *et al.* (1999) A
A (extremely symmetrical)	10 M	0.50	0.50	100.00	0.00	0.00
B	10 M	0.46	0.54	83.49	0.16	0.09
C	2 M + 3 S + 5 A	0.28	0.72	37.93	0.56	0.45
D	10 A	0.09	0.91	10.50	0.89	0.81
E	10 A	0.05	0.95	5.26	0.95	0.90
F (extremely asymmetrical)	10 A	0.00	1.00	0.00	1.00	1.00

* M = metacentric; S = submetacentric; A = acrocentric
