# Supplementary figures and images for: Intrachromosomal karyotype asymmetry in Orchidaceae
Source: Genet Mol Biol. 2017 Jun 22;40(3):610–9. doi: 10.1590/1678-4685-GMB-2016-0264 (PMC5596371; doi:10.1590/1678-4685-GMB-2016-0264)

**Figure S1** - UPGMA analysis using the intrachromosomal asymmetry values from TF% index.

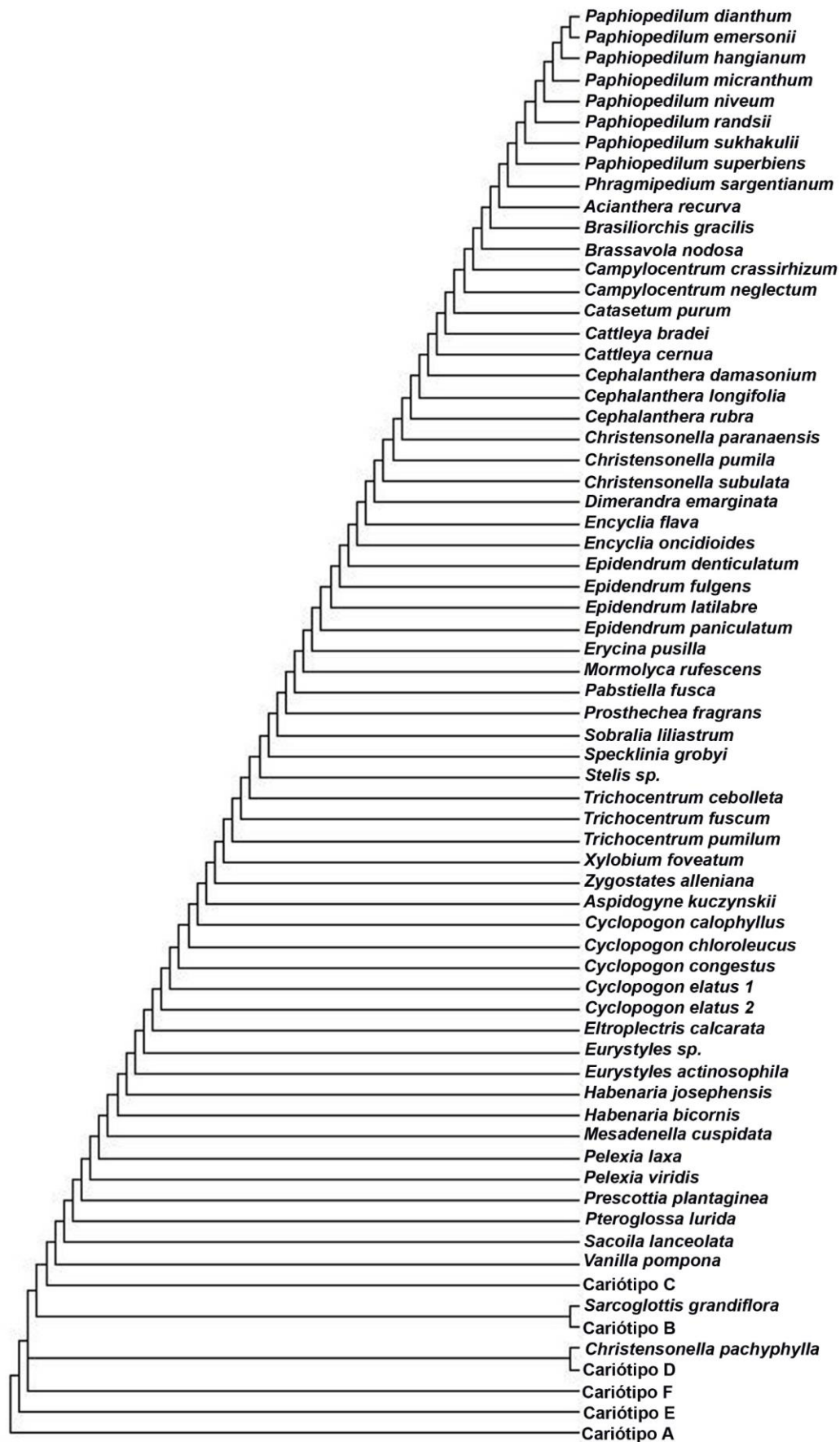

Supplement: Supplementary file 2 [file 1415-4757-gmb-1678-4685-GMB-2016-0264-Suppl02.pdf]

**Figure S3** - UPGMA analysis using the intrachromosomal asymmetry values from Syi and A indexes.

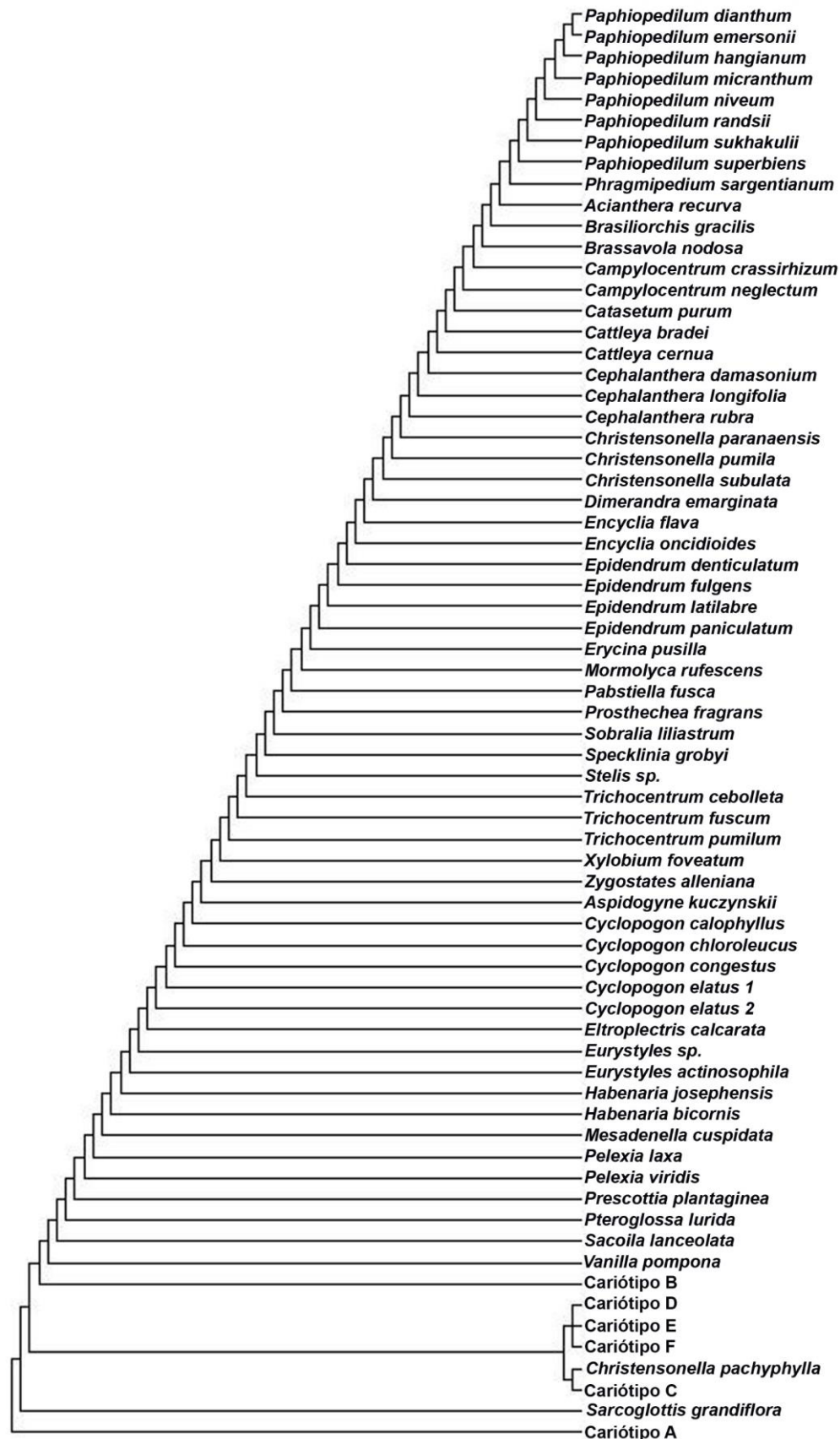

Supplement: Supplementary file 4 [file 1415-4757-gmb-1678-4685-GMB-2016-0264-Suppl04.pdf]
