# Supplementary material for: Intrachromosomal karyotype asymmetry in Orchidaceae
Source: Genet Mol Biol. 2017 Jun 22;40(3):610–9. doi: 10.1590/1678-4685-GMB-2016-0264 (PMC5596371; doi:10.1590/1678-4685-GMB-2016-0264)

Figure S2 - UPGMA analysis using the intrachromosomal asymmetry values from Ask% and A_1 indexes.

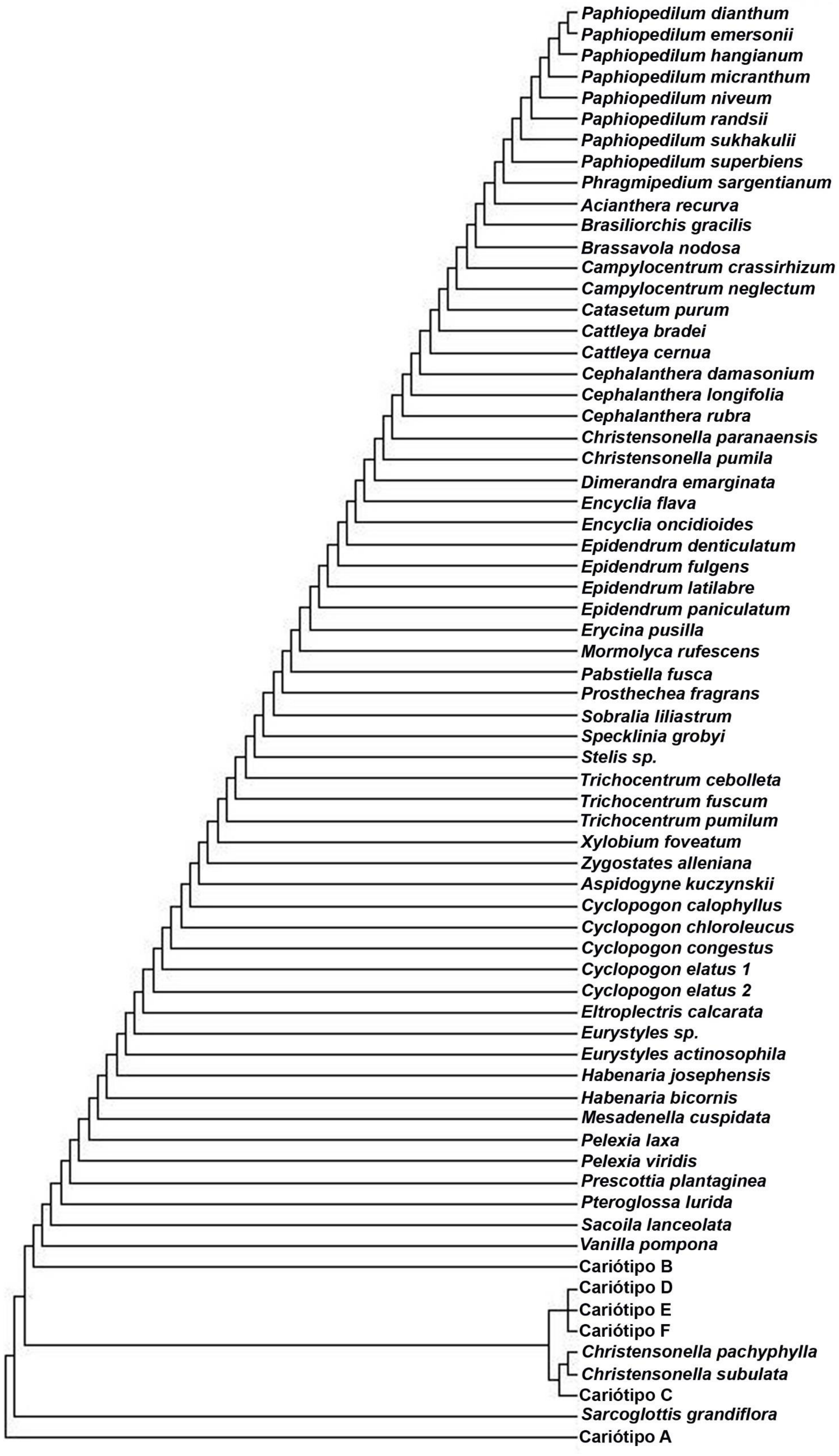

Supplement: Supplementary file 3 [file 1415-4757-gmb-1678-4685-GMB-2016-0264-Suppl03.pdf]
